# The Biopsychosocial Model in Type 2 Diabetes Mellitus: A Narrative Review

**DOI:** 10.7759/cureus.96814

**Published:** 2025-11-14

**Authors:** Yahia Khalil, Layan Abu-Khalaf, Raya Abu-Khalaf, Sandra Abu-Khalaf, Karrar Al-Balootti

**Affiliations:** 1 Internal Medicine, St Mary's Hospital, Isle of Wight NHS Trust, Newport, GBR; 2 Geriatric Medicine, Blackpool Victoria Hospital, Blackpool, GBR; 3 Medicine, Royal College of Surgeons in Ireland, Dublin, IRL; 4 Internal Medicine, Jordan Hospital, Amman, JOR; 5 Neurosurgery, Salford Royal NHS Foundation Trust, Manchester, GBR

**Keywords:** behavioural interventions, biopsychosocial model, glycaemic control, patient-centred care, type 2 diabetes mellitus

## Abstract

Type 2 diabetes mellitus (T2DM) is a major global health concern and a leading cause of morbidity and mortality. More than hundreds of millions of individuals are affected due to its rise at an alarming rate. Its prevalence is influenced by multiple factors, including socioeconomic status, biological and environmental factors. These factors interact in complex ways to determine the progression of the disease, its onset and outcomes in the long run. Socioeconomic status, particularly in low- and middle-income countries, in addition to gender differences, plays a significant role in disease burden. Nonetheless, behavioural patterns, such as poor diet and a sedentary lifestyle, impair glycaemic control. The chronic nature of T2DM causes a major psychological burden as it is also a leading cause of anxiety and depression, which negatively affects the patients’ ability to control their disease.

The biopsychosocial model provides a holistic framework that addresses these dimensions, supporting patients both mentally and physically. The framework demonstrates that the management of T2DM requires holistic treatments that address biological aspects, psychological elements and social factors. Treatment planning for T2DM should incorporate pharmacological management in addition to lifestyle changes that help patients become active participants in their healthcare. This review explores that psychosocial care integration into diabetes treatment leads to better patient adherence, improved blood sugar control and enhanced life quality. Additionally, it explores the biopsychosocial determinants of T2DM by emphasising their importance in the approach to a diabetic patient, which aids in bridging the existing gap between clinical treatment approaches and patients’ real-life experience to develop a targeted diabetes management strategy, enhancing patient-focused care for a T2DM patient.

## Introduction and background

Diabetes mellitus is a chronic metabolic condition characterised by elevated blood sugar levels [1]. There are different types of diabetes, which can be classified as type 1, type 2, pre-diabetes and gestational diabetes [2]. T2DM has the greatest prevalence, as it accounts for almost 90-95% of diabetes cases [3]. T2DM is characterised by a diminished insulin response, also known as insulin resistance [1]. Insulin is a hormone secreted by the pancreas, responsible for the regulation of glucose levels in the body. In the case of insulin resistance, insulin is not effective as the body is not using it properly, so insulin production rises to maintain glucose homeostasis [1]. Over time, insulin secretion declines, and the body stops responding to it, resulting in T2DM [2]. This happens due to the increased demand for insulin, which stimulates the pancreas to produce more insulin. In the long run, pancreatic beta cells that secrete insulin will be exhausted and damaged, leading to impeded secretion of that hormone [4]. 

T2DM may be symptomatic, but warning signs may be disregarded by the patient and therefore go unnoticed [5]. Having diabetes without early recognition can be dangerous, as delayed treatment and poor management can worsen the progression of the disease [5]. Therefore, it is important to understand what puts patients at risk, as this plays a role in early detection, hence a better prognosis. There are various known risk factors of T2DM. Obesity is considered the main risk factor for many non-communicable conditions such as cardiovascular disease, hypertension, and certain types of cancer [5]. It can also cause diverse psychological issues and physiological disabilities. Another risk factor that contributes to T2DM is genetics, as having a history of a family member (especially first-degree) diagnosed with diabetes increases the potential risk of having diabetes by two- to six-fold, as opposed to those without a previous family history [6]. Moreover, a sedentary lifestyle or physical inactivity can increase the risk of developing diabetes as it may lead to increased weight and obesity [7]. Also, people older than 45 years old are at a higher risk of developing T2DM, as increasing age will affect the body’s ability to maintain glucose levels [8]. Women with polycystic ovarian syndrome (PCOS) are more susceptible to T2DM, as indicated by Carreau and Baillargeon, who observed that 50% of women with PCOS develop T2DM by the age of 40 [9]. Ethnicity also contributes to a higher risk of diabetes, as people from African American, American Indian, Alaska Native, Latino and Hawaiian backgrounds are more likely to develop T2DM [10].

The initial step in the diagnosis of T2DM is taking a full history from the patient, which includes family, lifestyle, social, past medical and drug history. T2DM can be diagnosed by identifying the presence of hyperglycaemia in the patient and relevant blood tests. Hyperglycaemia symptoms include polyuria, polydipsia, unexplained weight loss, blurred vision and acanthosis nigricans (a skin condition causing dark pigmentation of skin folds, usually in the axilla, groin and neck) [1]. The latter is a sign of insulin resistance [1]. Other symptoms include itching around the head of the penis or vagina and delayed wound healing [5]. If T2DM is suspected in a symptomatic patient, only one abnormal glycated haemoglobin (HbA1c) or fasting plasma glucose level can be used, although repeated tests are recommended to confirm the diagnosis [11]. In asymptomatic patients, it is recommended not to rely on a single abnormal HbA1c or fasting plasma glucose result, as multiple tests must be done repeatedly, and it is preferred to do the same test [11]. Results of HbA1c of 48% mmol/mol or more (6.5%), a fasting plasma glucose level of 7 mol/L, and a random plasma glucose of 11.1 mol/L or more can all be indicative of persistent hyperglycaemia [11]. Diabetes can have several manifestations, including macrovascular and microvascular manifestations. Microvascular manifestations include neuropathy, nephropathy and retinopathy. Macrovascular complications include stroke, coronary heart disease and peripheral arterial disease [1]. Due to these complications, treatment at an early stage is essential. There are numerous treatments for T2DM, and a major part of the treatment is taking medications to help regulate blood sugar levels. The most common drug used for T2DM is a biguanide [12]. T2DM may develop over time and worsen, so doses of medications might be changed according to the severity of the disease. Insulin therapy is also used to manage hyperglycaemia, where insulin is given by one to three injections per day regimens [12]. A major side effect of insulin therapy is hypoglycaemia [12]. Diet and exercise can also significantly improve blood glucose levels and help in losing weight [13]. 

The aim of this review is to discuss the current evidence regarding the biopsychosocial framework in the approach to a T2DM patient. It highlights existing gaps in management approaches for diabetic patients and factors that contribute to poor adherence and worsen prognosis. Therefore, the review explores key recommendations to enhance patient-centred care for a diabetic patient by improving clinical practice, patient adherence, and quality of life. This narrative review was conducted using a search in PubMed, Scopus and Semantic Scholar, screening for peer-reviewed studies, review articles and regional reports published between 2015 and 2024. Keywords used included “type 2 diabetes mellitus,” “T2DM,” “biopsychosocial model,” “psychological determinants,” “behavioural factors,” and “social determinants of health.” We selected international literature reflecting global variations in psychosocial and behavioural aspects of T2DM. No formal inclusion or exclusion was applied due to the narrative design of the review.

## Review

Epidemiology

Diabetes is currently considered one of the main public health challenges worldwide. The prevalence of T2DM is growing quickly worldwide; in 2016, it was reported that 422 million adults live with diabetes (all types) around the globe [3]. During the previous two decades, the number of people with T2DM has doubled, as there are 4.7 million people diagnosed with diabetes in the United Kingdom (UK), with T2DM accounting for 90% of cases [14]. It is predicted that around 1,000,000 people have undiagnosed T2DM in the UK [15]. This can be considered a steep increase in the global prevalence of diabetes mellitus, as it rose from 4.7% in 1980 to 8.5% in 2014 [14]. In the UK, diabetes is considered one of the fastest-growing chronic conditions; this can be clearly noticed as a person is diagnosed with diabetes every two minutes, and one in 15 people has diabetes [14]. Diabetes UK predicts this figure will surpass 5.5 million by 2030, a more than two-fold increase over the past 20 years [14]. A study by Holden et al. [16] between 1991 and 2012, illustrated that the incidence of T2DM increased from 169 newly diagnosed people per 100,000 person-years (95% CI 160-178) to 515 (95 %CI 509-521) per 100,000 in 2010. Males and females had similar rates and patterns of T2DM, although males had a greater incidence rate than females. The trend of the incidence of T2DM can be linked to the increased consumption of unhealthy food and the lack of physical activity, thus resulting in an increased number of obese individuals [16]. 

Furthermore, an additional study conducted by Bagust et al. [17] aimed to calculate the incidence and prevalence rates of T2DM between 2000 and 2060. The study establishes that incident cases will peak around 2033 (228,000 per year), around 19% greater than it was in 2000 (192,500 per year). Whereas prevalent caseloads peak around 2036 (1,110,000 per year), 20% higher than starting numbers (926,000). Also, it has been reported that men have substantially faster relative growth rates than females (9.7% vs. 5.3% in 10 years for incidence, and 8.4% vs. 4.6% for prevalence) [17]. 

There are several research papers documenting how gender differences may affect the risk of having T2DM. These refer to biological variances between men and women, and are caused by variation in sex chromosomes, gene expression of autosomes and sex hormones, which can have various effects on body systems [18]. Investigations propose that visceral adiposity (or abdominal fat) is a strong risk factor for T2DM [19]. Central obesity can be linked to glucose intolerance and insulin resistance, which can dramatically increase the susceptibility to T2DM. Males were found to have more visceral fat than females, as females are more likely to exhibit gynoid adiposity (fat in hips and buttocks) [19]. A study was conducted to measure the relation between abdominal fat and the incidence of T2DM, where body mass index (BMI) and waist circumference were used to measure obesity. They discovered a strong association between abdominal fat and the incidence of T2DM, as shown by the obtained odds ratio of 2.14 (95% CI: 1.70-2.71; p < 0.0001) [19]. Additionally, it was evident that waist circumference provides equal or better predictive value than waist-to-hip ratio because central body fat increases risk by more than two times regardless of BMI [19]. This confirms that body-fat distribution changes rather than total body weight drive T2DM development according to gender-specific patterns of visceral fat accumulation [19].

The same modelling study from Bagust et al. [17] projected two main outcomes, which included rising disease numbers and healthcare costs. The research shows that diabetes-related complications together with healthcare expenses will reach their peak in 2045, up to 30% above current levels, while requiring 40-50% more National Health Service funding [17]. This demonstrates that the growing diabetes epidemic produces more than rising numbers of diagnosed patients because it affects older populations who survive longer with multiple chronic conditions [17].

The study by Kautzky-Willer et al. [18] demonstrated that T2DM global distribution patterns depend on sex and gender factors, which produce results that go beyond basic prevalence statistics. The study shows men develop T2DM at younger ages with lower body mass, while women experience a rapid rise in obesity-related diabetes after reaching middle age, and especially after menopause [18]. The study demonstrated that risk factors for T2DM differ between men and women because of how biological and social elements, including hormonal patterns, body fat distribution, economic status and mental stress, affect each sex. The research demonstrates that female obesity rates exceed male rates in areas with high gender inequality, yet men in wealthy nations develop metabolic problems before women do [18]. The existing gender differences between men and women require public health organisations to develop prevention programs which understand these differences.

Social determinants of T2DM

Social determinants of health are the conditions that establish the way people live and survive; these can be social - education, income and socioeconomic status - or physical - occupation, housing, ethnicity, gender and access to healthcare services [20]. These factors establish the non-medical elements which affect how people experience risk factors and their capacity to handle diabetes as a chronic disease. The unequal distribution of these determinants leads to major worldwide differences in disease prevalence and treatment results because low-income populations experience multiple forms of disadvantage in healthcare access, food security and educational opportunities [20]. According to several research papers, poverty, hunger, education and socioeconomic status are strongly linked to increased incidence, prevalence and burden of T2DM [21-23]. Hahn [20] demonstrated that social elements affect how biological sex characteristics manifest through their impact on eating patterns, stress reactions and medical treatment choices. People with lower educational levels tend to know less about diabetes warning signs, and they participate less in preventive care, which leads to delayed medical detection and disease advancement [21]. The combination of environmental and occupational factors which restrict physical activity and create dangerous living areas and block access to nutritious food leads to increased metabolic risk, which creates a continuous loop between social inequality and disease development [23]. Current literature recommends the use of lifestyle-changing techniques to maintain a healthy body weight and reduce body fat through lifestyle changes [21-23]. These include three main components, which are community-based education, dietary literacy promotion and environmental development to support physical activity and healthy food choices for all people, regardless of their financial situation or social status. The prevention and management of diabetes require targeted social determinant interventions to create equal opportunities for all people regardless of their socioeconomic status [20-23].

Socioeconomic status and inequality

Socioeconomic status is a descriptive term for the position of people in society based on occupation, education and economic aspects [21]. It was established that there is a strong correlation between socioeconomic status and the prevalence of T2DM, especially in low- and middle-income populations. A study in the UK showed that while T2DM is more common in deprived areas, type 1 diabetes showed no relation. This can be linked to Hart’s inverse law, which states that people who need healthcare the most are the least likely to receive it [24]. A Canadian study showed that T2DM is four times more prevalent in the lowest-income populations than in the highest-income populations [25]. This demonstrates the existence of an inversely proportional relation between socioeconomic status and T2DM risk, as the prevalence of the disease increased as income decreased. This can be explained by the fact that people with low socioeconomic conditions may suffer more from other associated risk factors of diabetes, such as depression, physical inactivity and poor dietary awareness, leading to increased weight and obesity. In addition, people from deprived areas tend to struggle following a healthy diet as unhealthier options offer more convenience at lower costs [22]. Also, this subgroup fails to participate in physical exercise as accessibility to fitness centres and such venues is extremely limited. Patients also find it difficult to access proper healthcare services and are left without appropriate treatments [21]. This may trigger unhealthy weight gain and unhealthy habits such as smoking and excessive alcohol consumption, which will lead to worse outcomes. Moreover, systematic review studies were conducted on inequalities in healthcare among T2DM patients by socioeconomic status and regional deprivation [26]. The results disclose the existence of noticeable inequalities, as it showed a clear trend of worse healthcare for low socioeconomic status patients [26]. These factors, in combination with reduced access to better healthcare and fitness venues, result in increased co-morbidities such as hypertension and obesity, ultimately increasing the risk of developing diabetes [26].

The relationship between deprivation and T2DM prevalence remains evident, yet socioeconomic status affects how people eat and live their lives and how their bodies metabolise substances over time. People from lower socioeconomic backgrounds struggle to follow a healthy diet because they lack access to fresh produce and must rely on affordable, high-calorie foods [21]. The restricted food choices lead to ongoing high blood sugar levels and weight gain, which creates a continuous pattern between poverty and metabolic diseases [21]. People who work long hours and experience stress and lack social support tend to avoid physical exercise because of their unstable work situation and low-income status [22].

Walker et al. [23] demonstrated that social determinants of health, including education level and neighbourhood environment, and healthcare access, determine diabetes management and complications better than biomedical factors. The social status of people determines their healthcare results according to Barnard-Kelly and Cherñavvsky [24], who showed that disadvantaged socioeconomic groups receive delayed medical diagnoses and poor blood sugar control and experience more complications, which leads to ongoing health disparities. The Canadian Community Health Survey [25] demonstrated that T2DM prevalence rises consistently with income level until it reaches four times higher in the lowest income group than in the highest income group. 

People from disadvantaged socioeconomic backgrounds face worse diabetes self-management due to stress, poor health understanding and reduced confidence in their abilities [26]. The combination of psychological stress factors makes people less likely to follow their diet plans and medication schedules while preventing them from seeking medical care and increasing their risk of developing preventable habits like smoking and sedentary behaviour [26]. The evidence shows that socioeconomic position affects T2DM development risk and disease progression and treatment results through multiple social, psychological and behavioural factors [23-26]. 

Age and gender

In the past few decades, T2DM was more common among adults, but now it also affects children and teenagers. This is due to the rising prevalence of obesity in young people, especially in high-income countries [27]. It is important to consider the age of onset of T2DM as it can be an indicator of many features of the disease. Research on Diabetes in Youth established that T2DM presenting at a younger age is often more aggressive and can lead to more serious morbidities and eventually mortality [28]. In addition, it was found that T2DM patients diagnosed below the age of 40 have the highest risk of all-cause mortality, whereas patients diagnosed after the age of 80 have no additional mortality risks [27]. The existence of a reversed relationship between the age of onset of diabetes and complication risk and death can be linked to increased lifetime exposure to hyperglycaemia, which adversely impacts body systems, leading to higher co-morbidity risk and poorer prognosis. 

Gender differences in glycaemic control have been detected, and it was found that women have better blood sugar levels compared to men [29]. Roy et al. [29] identified metabolic and behavioural parameters related to effective glycaemic management in a retrospective study of 263 individuals. Males were 53.6% of all patients, while females made up 46.4%. 66.4% of males showed an inadequate T2DM control, with an average HbA1c of 8.87%. Nevertheless, 56.9% of the female gender had optimal control, with an average HbA1c of 6.24% [29]. Females were less likely to receive help and support, faced more barriers, had less self-efficacy and lower levels of diabetes self-management, resulting in poor glycaemic control [26]. This can be clarified by disclosing the association of cultural and social norms, as these norms can be something like gender roles and the importance of family.

Research findings demonstrate that the age when someone receives their T2DM diagnosis determines their disease progression [27]. The study by Sattar et al. [27] showed that people who receive their diabetes diagnosis before age 40 develop microvascular and macrovascular complications at an earlier stage and face increased lifetime cardiovascular risk than those diagnosed later in life. The study by Ang [28] shows that people with early-onset diabetes experience longer periods of insulin resistance and oxidative stress, which leads to faster development of endothelial damage, nephropathy and neuropathy. The research indicates that public health programs need to focus on detecting diabetes early in young people to reduce the total amount of disease they will develop [28]. The risk factors for diabetes development differ between men and women because men develop diabetes at lower body mass indices and show more visceral fat, but women experience more severe metabolic effects when their disease becomes active [29]. The combination of age and gender affects disease progression because it involves hormonal changes and body composition differences, and behavioural and psychosocial elements that affect when people develop diabetes and how their condition advances and ends [29].

Ethnicity

Ethnicity is a multifaceted construct that groups people who identify with one another through shared cultural traditions. The UK is known for its wide diversity of ethnicities, as the most recent Census of England and Wales, 14% of the population claimed to be of minority ethnic origin [30]. London “SABRE” states that 40-50% of South Asian and African-Caribbean men and women would have T2DM by the age of 80, which is at least twice the proportion of their age-matched white European peers [30]. A quicker development of T2DM is particularly apparent; a recent review of UK primary care data found that the average age of diagnosis among South Asian and black African or Caribbean people is 10-12 years younger than in white Europeans [30]. 

Socioeconomic status and ethnicity are significant predictors of diabetes care disparities. Individuals in the most impoverished quintile of socioeconomic status and from ethnic minority groups had less monitoring of HbA1c, estimated glomerular filtration rate, retinopathy, and neuropathy, while there was no disparity in monitoring of blood pressure [31]. 

The study by Goff [30] shows that T2DM affects different ethnic groups through distinct disease mechanisms because South Asians develop insulin resistance and liver fat at lower body mass indices than white Europeans. The current diagnostic standards fail to detect sufficient risk in these populations, which results in delayed medical detection and inadequate prevention methods [30]. The combination of dietary patterns that include refined carbohydrates, decreased physical activity, and urbanisation makes ethnic minority groups more susceptible to disease development [30]. The transition of migrant populations to Westernised lifestyles results in faster development of diabetes complications and more severe diabetes symptoms [30].

The study by Whyte et al. [31] shows that healthcare disparities based on ethnicity affect both disease tracking and treatment results in patients. The research shows that minority patients received fewer combination therapies and failed to reach their blood sugar targets even though their medical conditions were equivalent [31]. The ongoing structural barriers in diabetes treatment continue to exist because patients face multiple barriers that prevent them from receiving proper medical care [31]. Public health programs need to develop specific strategies which focus on risk factors unique to different ethnic groups, enhance screening for at-risk populations and deliver equal healthcare access to reduce T2DM disparities [30,31].

Behavioural and psychological interventions

Consistent with what we discussed previously, T2DM is highly associated with poor diet, unhealthy habits and lack of physical activity. Behavioural interventions aim to modify and improve patients’ self-management manners, which will help them find suitable coping mechanisms with the disease. Behavioural change techniques (BCTs) aim to help people achieve a healthier diet and better lifestyle by supporting patients’ behavioural changes. These modifications are increasing physical activity, following a healthy diet, smoking cessation, weight loss and finding better coping mechanisms [32]. It was found that multiple BCTs implemented in T2DM were linked to clinically noteworthy changes in HbA1c and reduced blood glucose levels [33]. BCTs include techniques such as instructing people on how to perform a behaviour, practising it and then demonstrating it to act [32]. This led to a noticeable reduction in HbA1c levels by having a positive effect on dietary and physical activity behaviours. According to Cradock et al. [32], these three main BCTs were found to be more effective when delivered together rather than isolated, as they work synergistically. Furthermore, it was found that diet and physical activity interventions delivered by experts such as exercise physiologists and dietitians through face-to-face sessions might be the most effective method to bring those interventions. It is important to mention that men were found to receive more benefit from one-to-one classes rather than groups, whereas women achieved better glycaemic control and more efficient behavioural changes through group sessions [32]. This can be indicative of the natural differences between the two genders, as females tend to be more sociable and like interacting with others, as they consider it motivational. It was also shown that more frequent physical contact and physical activity under supervision may enhance the effectiveness of those techniques [32]. Other effective behavioural interventions were problem solving, feedback on behaviour, adding environmental objects and social comparison. BCTs targeting dietary habits changes were linked to more than 0.3% (3.3 mmol/mol) fall in HbA1c (95% CI -0.40, -0.23) [32]. However, meta-analysis revealed that studies that aim to modify dietary environment showed a more significant reduction in HbA1c levels, as it caused about a 0.5% reduction (5.5 mmol/mol) (95% CI -0.65, -0.34) [32]. These data imply that modifying or managing dietary environmental factors, rather than dietary behaviour change, may be more helpful in lowering HbA1c in patients with T2DM. Another study included low carbohydrate (n = 9), low fat (n = 16), high protein (n = 5), meal replacements (n = 4), low glycaemic index (n = 3), medical nutritional treatment (n = 2), Mediterranean (n = 2) and others (n = 13) were among the dietary methods studied in this review. The highest decreases in body weight and HbA1c were found in studies employing meal replacements and high-protein diets (0.56% (6.2 mmol/mol) and 0.5% (5.5 mmol/mol), respectively) [32]. In this meta-analysis, there was a control and intervention group to compare the outcomes of the study, which showed noticeable differences between the groups over variable time points: 0-3, 3-6, 6-12 and 12-24 months. The difference in body weight loss between intervention and control groups was 2.34 kg (95% CI -2.99, -1.69; p < 0.00001) at zero to three months, 2.94 kg (95% CI -3.92, -1.97; p < 0.00001) at three to six months, 2.27 kg (95% CI -3.32, -1.21; p < 0.0001) at six to 12 months, and 2.14 kg (95% CI -3.34, -0.93; p = 0.0005) at 12-24 months [32]. A reduction in body weight of 2.41 kg (95% CI 2.96-1.86; p < 0.00001) was seen when all studies and time points were combined [32]. HbA1c levels were reduced the most with high-protein diets and meal replacement programmes. When all dietary regimens were integrated in meta-analyses, a clinically significant difference in HbA1c was identified at zero to three, three to six, and six to 12 months. Weight reduction did occur between 12 and 24 months; however, it slowed down with time. From the above study, we can see that BCTs that aim to change dietary habits by modifying nutritional features showed a beneficial influence, helping participants lose weight and reduce body fat. This will aid in achieving well-controlled glycaemia, reducing the risk of developing T2DM.

Exercise is another effective non-pharmacological therapeutic regimen for T2DM. High to moderate physical activity has the potential to prevent cardiovascular events and reduce the risk of mortality in patients. Moderate physical activity assists diabetic patients in managing their weight and enhances better glycaemic control [32]. The American Diabetes Association and the UK Chief Medical Officer stated that to minimise the risk of cardiovascular disease and T2DM, adults should engage in at least 150 minutes of moderate-intensity physical exercise (or at least 60-90 minutes of high-intensity exercise) every week [34]. According to Helmrich et al. [35], men with the highest risk of diabetes, such as those with a high BMI, a history of hypertension, or offspring of diabetic parents, benefited the most from physical activity. Moreover, it was concluded that physical activity may have a larger protective impact in reducing the incidence of diabetes in obese or other diabetes-prone individuals. BCTs that aim to change dietary environment and adjust nutritional habits were found to be more effective in reducing body fat, therefore achieving healthier glycaemic control and reducing the risk of having diabetes [35]. Furthermore, increased physical activity was also associated with many beneficial consequences, as it helps patients lose weight. However, BCTs targeting dietary habits were found to be scientifically proven to reduce HbA1c levels more than high physical activity and exercise. This was due to a lack of research on the importance of physical activity and its impact on blood glucose levels. 

Recommendations for adopting the biopsychosocial model in T2DM

Although pharmacological advances have improved glycaemic control, the primary focus and approach to T2DM patients remains biomedical, which undermines the importance of psychosocial and behavioural dimensions affecting outcomes and worsening the epidemiological burden [7,13,15]. Patients with a lower socioeconomic status have limited access to healthcare, and are less knowledgeable about their condition, which leads to higher complications, as in line with the inverse care law [24-26]. Stress-management interventions are another essential aspect that influences long-term adherence; hence, it is imperative that the approach to T2DM integrates psychosocial care [32-34]. Furthermore, patient preferences and motivational readiness are often not taken into account in care decisions, leading to fragmented management and reduced engagement [36]. The biopsychosocial model application in T2DM moves healthcare from biomedical-only treatment to complete holistic health approaches, which focus on patient needs and use multiple disciplines and behavioural methods. The recommendations combine data from multiple assessment which studies physical elements, mental aspects and social factors [36-39]. The current research evidence supports this change through studies to create a usable method for delivering biopsychosocial care to T2DM patients [36-39].

Adopt Patient-Centred and Shared Decision-Making Frameworks

The current guidelines show that patient-centred care stands as the essential base for biopsychosocial treatment of diabetes [36]. The approach of shared decision-making enables healthcare providers to merge treatment plans with individual patient preferences, emotional readiness and cultural background information. The decision cycle framework developed by Hsu et al. [36] helps patients maintain active involvement in setting goals, evaluating and adjusting their treatment plans. The collaborative approach between patients and healthcare providers leads to better treatment compliance and stronger self-confidence while making management plans that match individual psychological needs together as part of coordinated multidisciplinary teams [36].

Embed Multidisciplinary and Coordinated Care Teams

The treatment of T2DM needs healthcare providers to work together as part of coordinated multidisciplinary teams. The research by Loveman et al. [37] shows that healthcare teams which include physicians, nurses, pharmacists and dietitians produce better blood sugar control and patient contentment results. The team-based approach to care delivery combines medical treatment with dietary advice, mental health support and social network connections [37]. The practice of team-based care through shared documentation and regular team meetings helps prevent care fragmentation, which supports patients' long-term involvement in their treatment.

Personalise and Individualise Treatment and Address Psychosocial Readiness

The process of individualising treatment stands as a fundamental requirement for delivering effective biopsychosocial care. The evaluation process proposed by Hsu et al. [36] includes metabolic parameter assessment together with motivational state evaluation, stress level assessment and family support evaluation before starting therapy escalation. The treatment approach of Davies et al. [38] combines disease severity assessment with the patient's ability to handle complex medication plans. The approach of matching interventions to individual psychosocial readiness levels helps doctors avoid treatment delays while creating less patient distress and enabling disease management to match personal life experiences [36,38].

Prioritise Behavioural and Lifestyle Interventions

The approach to diabetes management under a biopsychosocial framework depends on lifestyle changes. The research by Nor et al. [39] shows that behavioural interventions based on the Transtheoretical Model allowed patients to follow treatment plans better because they align with their stage of change. This combination of behavioural counselling with motivational interviewing and goal-setting techniques enables patients to develop lifestyle changes that continue after clinical support ends. This helps achieve tight glycaemic control and improved psychosocial support for patients. 

Reinforce Education and Continuous Follow-Up

The maintenance of biopsychosocial integration depends on ongoing patient education. The research by Loveman et al. [37] and Davies et al. [38] demonstrates that patients who receive appropriate educational programs achieve better metabolic results and develop greater self-empowerment. The educational content should move past basic disease information to teach patients effective coping methods, stress management techniques and social network utilisation skills. Patients who receive ongoing care through community-based services or telehealth platforms maintain their learning progress while staying accountable to their treatment plan and receiving emotional support, which enables them to develop lasting self-care abilities.

## Conclusions

T2DM is a chronic disease that requires exploration using the biopsychosocial model. T2DM has biological, behavioural, and psychological effects on the patient, and it is not only manifested with somatic symptoms but also with psychological changes. T2DM patients typically present with poor diet and lack of physical exercise, which worsens the condition. The biopsychosocial model aims to prevent and treat disease through both physical and psychological interventions. Similarly, BCTs are used to help the patients modify food habits and encourage them to engage in increased physical activity by addressing both physical and mental behaviour. These interventions can reduce HbA1c levels and thereby achieve better glycaemic control. To achieve optimal diabetes management, a patient-focused approach is required, which unites behavioural interventions with educational programs and psychosocial support services. The proposed recommendations for structured decision-making, lifestyle modification and continuous education will connect pharmacological treatment to patient experiences, thus addressing current care deficiencies. The biopsychosocial framework produces better clinical results while simultaneously improving life quality and enduring adherence in T2DM patients.
